# Supplementary material for: An agricultural digital twin for mandarins demonstrates the potential for individualized agriculture
Source: Nat Commun. 2024 Feb 20;15:1561. doi: 10.1038/s41467-024-45725-x (PMC10879191; doi:10.1038/s41467-024-45725-x)
Supplement: Supplementary file 1 — Supplementary Information [file 41467_2024_45725_MOESM1_ESM.pdf]

Supplementary Information

An agricultural digital twin for mandarins demonstrates the potential for individualized agriculture

Steven Kim[1] and Seong Heo[2*]

[1]Department of Mathematics and Statistics, California State University, Monterey Bay, Seaside 93955, USA
[2]Department of Horticulture, Kongju National University, Yesan 32439, Republic of Korea
[*]Correspondence: Seong Heo (heoseong@kongju.ac.kr)

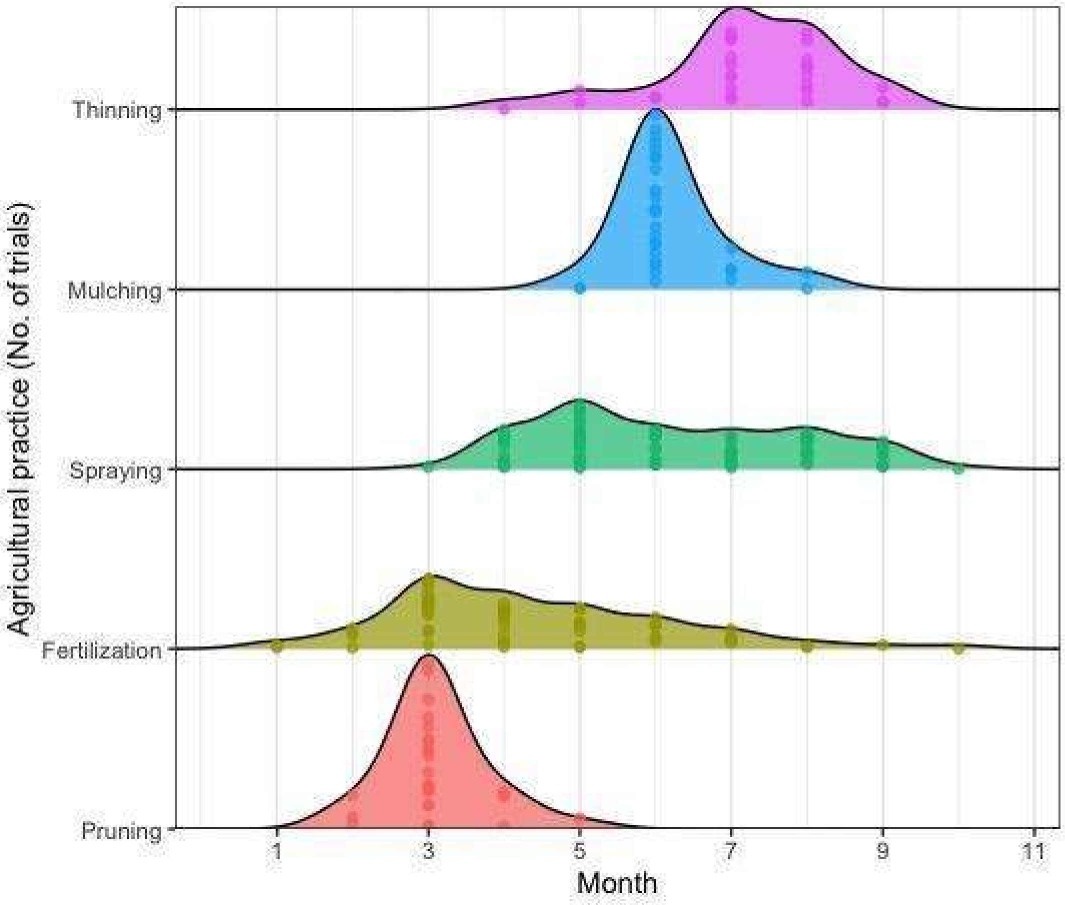

Supplementary Fig. 1. Frequencies of the five agricultural practices recorded from January to October in 2021 for 27 orchards. Source data are provided as a Source Data file.

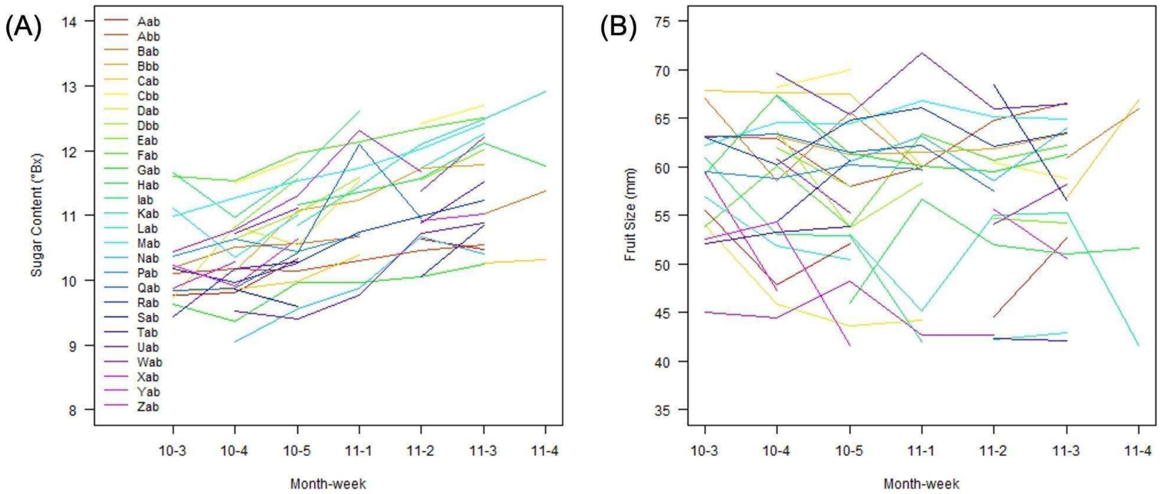

Supplementary Fig. 2. Average trend of sugar content (Panel A) and fruit size (Panel B) by orchard between mid-October (the third week of October) and late November (the fourth week of November). A total of *N* = 39,679 mandarins in the 27 orchards are used for this graphic. Source data are provided as a Source Data file.

Supplementary Table 1. Estimated parameters for the fixed-effect (harvest week), standard errors (SE), degrees of freedom (DF), t-values (t), and p-values (p). The p-values are two-sided and calculated using the t test with the degrees of freedom estimated by the Satterthwaite's method under the mixed-effects model for sugar content and fruit size with orchards as random-effects. The reported p-values are adjusted using the method of Benjamini & Yekutieli (2001)[53]. The p-values are reported using the scientific notation because they are very small, and a p-value is indicated by <1e-100 when it is less than 1e-100. A total of 39,645 mandarins from 2,693 trees in the 27 orchards were included in the analysis.

	Parameter	Estimate	SE	DF	t	p
Sugar content[a]	Intercept	10.094	0.134	28	75.053	–
	Month-week (10-4)	0.309	0.029	39627	10.523	2.28e-25
	Month-week (10-5)	0.308	0.029	39632	10.779	1.60-e26
	Month-week (11-1)	0.658	0.028	39628	23.745	<1e-100
	Month-week (11-2)	1.047	0.030	39628	34.850	<1e-100
	Month-week (11-3)	1.248	0.029	39632	42.942	<1e-100
	Month-week (11-4)	1.435	0.029	39632	49.116	<1e-100
Fruit size[b]	Intercept	60.958	1.287	27	47.360	–
	Month-week (10-4)	–2.641	0.155	39617	–17.076	1.58e-64
	Month-week (10-5)	–2.505	0.151	39619	–16.632	2.49e-61
	Month-week (11-1)	–4.527	0.146	39618	–30.997	<1e-100
	Month-week (11-2)	–3.700	0.158	39617	–23.343	<1e-100
	Month-week (11-3)	–5.001	0.153	39619	–32.624	<1e-100
	Month-week (11-4)	–4.724	0.154	39619	–30.665	<1e-100

[a]The variances associated with the orchard-level random-effect and unexplained random error were estimated as 0.4712 and 1.1272, respectively.

[b]The variances associated with the orchard-level random-effect and unexplained random error were estimated as 44.25 and 31.34, respectively.

Supplementary Table 2. Estimated parameters for the fixed-effect (harvest week), standard errors (SE), degrees of freedom (DF), t-values (*t*), and *p*-values (*p*). The p-values are two-sided and calculated using the t test with the degrees of freedom estimated by the Satterthwaite's method under the mixed-effects model for sugar content and fruit size with orchards and trees as random-effects. The reported p-values are adjusted using the method of Benjamini & Yekutieli (2001)[53]. The p-values are reported using the scientific notation because they are very small, and a p-value is indicated by <1e-100 when it is less than 1e-100. A total of 39,645 mandarins from 2,693 trees in the 27 orchards were included in the analysis.

	Parameter	Estimate	SE	DF	*t*	*p*
Sugar content[a]	Intercept	10.109	0.133	27	75.859	–
	Month-week (10-4)	0.314	0.022	36991	14.575	2.48e-47
	Month-week (10-5)	0.311	0.021	36981	14.829	6.54e-49
	Month-week (11-1)	0.658	0.020	36977	32.311	<1e-100
	Month-week (11-2)	1.049	0.022	36977	47.486	<1e-100
	Month-week (11-3)	1.252	0.021	36986	58.562	<1e-100
	Month-week (11-4)	1.439	0.021	36988	66.966	<1e-100
	Position (low)	–0.039	0.010	36965	–4.012	2.51e-04
	Position (middle)	–0.020	0.010	36965	–2.122	0.131
Fruit size[b]	Intercept	60.991	1.288	27	47.363	–
	Month-week (10-4)	–2.641	0.155	39615	–17.076	2.29e-64
	Month-week (10-5)	–2.505	0.151	39617	–16.631	3.62e-61
	Month-week (11-1)	–4.527	0.146	39616	–30.996	<1e-100
	Month-week (11-2)	–3.700	0.158	39615	–23.342	<1e-100

	Month-week (11-3)	–5.001	0.153	39617	–32.624	<1e-100
	Month-week (11-4)	–4.724	0.154	39617	–30.665	<1e-100
	Position (low)	–0.017	0.069	39610	–0.244	1.000
	Position (middle)	–0.082	0.069	39610	–1.186	0.849

[a]The variances associated with the orchard-level random-effect, tree-level random-effect, and unexplained random error were estimated as 0.4643, 0.5070, and 0.6088, respectively.

[b]The variances associated with the orchard-level random-effect, tree-level random-effect, and unexplained random error were estimated as 44.25, <0.01, and 31.34, respectively.

Supplementary Note 1. README

Section 1. System requirements

All software dependencies and operating systems (including version numbers): Users must install R and RStudio in order to run the codes provided in the GitHub and Zenodo repository. R and RStudio run on both Windows and MacOS. R must be installed, then RStudio can be installed. See Section 2 (Installation Guide) for installing R and RStudio.

Versions the software has been tested on: R version 4.3.1.

Any required non-standard hardware: None

Section 2. Installation guide

Instructions: See here. R must be installed, then RStudio can be installed. All R packages can be installed in RStudio. See here for installing packages. No installation is needed for the agricultural DT demo.

Typical install time on a "normal" desktop computer: Installing R and RStudio should take less than 5 minutes each. (It may depend on internet speed.) Installing a package in RStudio usually takes less than one minute (or a few seconds) depending on size and dependencies.

Section 3. Demo

Instructions to run on data: Agricultural DT demo can be used as follows.

1. Use the first dropdown menu to select an orchard.
2. Click on the Submit button below the dropdown menu. The second dropdown menu will be updated (trees in the selected orchard).
3. Use the second dropdown menu to select a tree.
4. Click on the Submit button below the dropdown menu. After a few seconds (less than 3 seconds), results will show in the panels on the right side.

Expected output: When an orchard is selected, the most recent information is presented in the following tabs:

1. Map: This tab shows the map of Jeju Island and the location of an orchard selected
2. Soil: This tab shows the distribution of observed soil chemical property values (available phosphorus, exchangeable potassium, calcium, and magnesium, acidity, organic matter, and electrical conductivity) in Jeju Island with the percentile (at the selected orchard) and the RDA recommendation (shaded in gray).
3. Weather: This tab shows the distribution of observed temperature, humidity, and air pressure in orchards in Jeju Island with the percentile (at the selected orchard).

4. Agricultural Practice: This tab shows the frequency of agricultural practices (fertilization, mulching, spraying, pruning, and thinning) at the selected orchard and compares with orchards in Jeju Island.
5. Sugar Content Distribution: This tab shows the inter-orchard and intra-orchard distributions of sugar content (°Bx). It is based on the recent week in the data. The inter-orchard distribution represents mandarins by orchards in the week, and the intra-orchard distribution represents mandarins by trees in the selected orchard in the week.
6. Fruit Size Distribution: This tab shows the inter-orchard and intra-orchard distributions of fruit size (mm). It is based on the recent week in the data. The inter-orchard distribution represents mandarins by orchards in the week, and the intra-orchard distribution represents mandarins by trees in the selected orchard in the week.
7. Sugar Content History: This tab shows longitudinal patterns of the weekly average sugar content (°Bx). The orchard-level figure shows the selected orchard and all other orchards in Jeju Island. The tree-level figure shows the selected tree and all other trees in the selected orchard.
8. Fruit Size History: This tab shows longitudinal patterns of the weekly average fruit size (mm) at orchard-level and tree-level in the selected orchard. The orchard-level figure shows the selected orchard and all other orchards in Jeju Island. The tree-level figure shows the selected tree and all other trees in the selected orchard.

Expected run time for demo on a "normal" desktop computer: A few seconds (less than 3 seconds)

Section 4. Instructions for use

How to run the software on your data: See Section 3 (Demo).

Reproduction instructions: For reproducing figures and results presented in the manuscript, all source codes are provided in the GitHub and Zenodo repository. Make sure RStudio and relevant packages are installed before attempt.
