# Supplementary material for: An agricultural digital twin for mandarins demonstrates the potential for individualized agriculture
Source: Nat Commun. 2024 Feb 20;15:1561. doi: 10.1038/s41467-024-45725-x (PMC10879191; doi:10.1038/s41467-024-45725-x)
Supplement: Supplementary file 2 — Description of Additional Supplementary Files [file 41467_2024_45725_MOESM2_ESM.pdf]

File Name: Supplementary Data 1
Description: Data being merged to create Fig. 1.
(1-1) File that merges soil chemical properties, regional codes, and addresses.
(1-2) Coordinates (latitude, longitude) data based on address through geocoding.
(1-3) File that merges geocoding results and soil chemical properties.
(1-4) File combining sugar content and fruit size and coordinates (latitude and longitude) of 30 selected orchards.
(1-5) Chemical properties data of orchard soil in Jeju Island.

File Name: Supplementary Data 2
Description: File that merges geocoding results and soil chemical properties.

File Name: Supplementary Data 3
Description: Data being merged to create Fig. 3.
(3-1) File that merges geocoding results and soil chemical properties.
(3-2) Fruit data from 30 selected orchards.
(3-3) Fruit data from 3 replicates investigated by location for 30 selected orchards.
(3-4) Environmental data from 30 selected orchards.
(3-5) Agricultural practice data from 30 selected orchards.

File Name: Supplementary Data 4
Description: Data being merged to create Fig. 4.
(4-1) File that merges geocoding results and soil chemical properties.
(4-2) Fruit data from 30 selected orchards.
(4-3) Fruit data from 3 replicates investigated by location for 30 selected orchards.
(4-4) Environmental data from 30 selected orchards.
(4-5) Agricultural practice data from 30 selected orchards.

File Name: Supplementary Data 5
Description: Data being merged to create Fig. 5.
(5-1) Fruit data from 3 replicates investigated by location for 30 selected orchards.
(5-2) Fruit grade data from 30 selected orchards.
(5-3) Agricultural practice data from 30 selected orchards.
(5-4) Address and coordinate information for 30 selected orchards.
(5-5) Soil chemical properties data among the 30 selected orchards.

File Name: Supplementary Data 6
Description: Data being merged to create Fig. 6.
(6-1) File that merges geocoding results and soil chemical properties.
(6-2) Fruit data from 30 selected orchards.

(6-3) Fruit data from 3 replicates investigated by location for 30 selected orchards.
(6-4) Environmental data from 30 selected orchards.
(6-5) Agricultural practice data from 30 selected orchards.

File Name: Supplementary Data 7
Description: Fruit and environmental data from 30 selected orchards for machine learning analysis.

File Name: Supplementary Data 8
Description: Fruit data from 3 replicates investigated by location for 30 selected orchards.

File Name: Supplementary Data 9
Description: Data being merged to create Fig. 9.
(9-1) File that merges geocoding results and soil chemical properties.
(9-2) Fruit data from 30 selected orchards.
(9-3) Fruit data from 3 replicates investigated by location for 30 selected orchards.
(9-4) Environmental data from 30 selected orchards.
(9-5) Agricultural practice data from 30 selected orchards.

File Name: Supplementary Data 10
Description: Data for Supplementary Figure 1. Agricultural practice data from 30 selected orchards.

File Name: Supplementary Data 11
Description: Data for Supplementary Figure 2.
(11-1) A file that merges geocoding results and soil chemical properties.
(11-2) Fruit data from 30 selected orchards.
(11-3) Fruit data from 3 replicates investigated by location for 30 selected orchards.
(11-4) Environmental data from 30 selected orchards.
(11-5) Agricultural practice data from 30 selected orchards.

File Name: Supplementary Data 12
Description: Raw GIS data including soil chemical properties and regional code. The Korean language pack is needed to read the Korean fonts (addresses recorded in Korean), but the Korean fonts are not necessary for the relevant data analysis.
